# Quality of Life and Job Satisfaction Among Nurses and Physicians in Greek Public Hospitals: Associations with Protocol-Based Work and Workplace Characteristics

**DOI:** 10.3390/healthcare14182932

**Published:** 2026-09-09

**Authors:** Katerina Kloumpa, Eleni Lahana, Eleni Albani, Christos Kleisiaris, Georgios Manomenidis, Georgios Dentsikas, Stiliani Kotrotsiou, Nikolaos Bakalis

**Affiliations:** 1 Department of Nursing, Faculty of Health and Rehabilitation Sciences, University of Patras, 26504 Patras, Greece; katerinakloumpa75@yahoo.com (K.K.); ealbani@upatras.gr (E.A.); stkotrots@upatras.gr (S.K.); 2 Department of Public and One Health, Faculty of Health Sciences, University of Thessaly, 43100 Karditsa, Greece; elahana@uth.gr; 3 Department of Nursing, Faculty of Health Sciences, University of Thessaly, 41500 Larisa, Greece; kleisiaris@uth.gr; 4 Department of Nursing, Faculty of Health Sciences, Democritus University of Thrace, 68100 Alexandroupolis, Greece; gmanomen@nurs.duth.gr; 5 6th Health Region of Peloponnese, Ionian Islands, Epirus and Western Greece, 26441 Patras, Greece; g.dentsikas@dypede.gr

**Keywords:** nurses, physicians, quality of life, job satisfaction, protocol-based work, public hospitals

## Abstract

**Highlights:**

**What are the main findings?**

- Vitality and general health were the lowest-scoring dimensions of health-related quality of life among both nurses and physicians. Compared with physicians, nurses reported significantly higher levels of mental health, social functioning, and job satisfaction.
- Protocol-based work was consistently associated with higher job satisfaction and better scores in several HRQoL dimensions among both nurses and physicians, although causal relationships cannot be inferred from this cross-sectional study.

**What are the implications of the main findings?**

- Protocol-based work may represent a potentially modifiable organizational characteristic associated with nurses’ and physicians’ quality of life and job satisfaction during public health emergencies and other periods of heightened clinical demand.
- The findings support further evaluation of organizational strategies, including protocol implementation and workload management, as potentially relevant factors in workforce wellbeing.

**Abstract:**

**Background:** Health-related quality of life (HRQoL) and job satisfaction are important indicators of nurses and physicians’ wellbeing and system sustainability, particularly under conditions of high clinical demand. Although organizational determinants of nurses and physicians’ wellbeing have received increasing attention, evidence regarding protocol-based work as a specific organizational characteristic remains limited. **Methods:** A multicenter cross-sectional study was conducted in 11 Greek public hospitals between September 2022 and March 2023. The study included 405 nurses and physicians (214 nurses and 191 physicians). HRQoL was assessed with the SF-36 and job satisfaction with the MSQ-Short Form. Protocol-based work was assessed using a single self-reported dichotomous item referring to the use of standardized clinical protocols during the pandemic. Associations between workplace characteristics and outcomes were examined using multivariable linear regression models. **Results:** Vitality (57.24 ± 17.61 among nurses and 53.92 ± 16.93 among physicians) and general health (56.58 ± 19.81 and 53.42 ± 16.32, respectively) were the lowest-scoring HRQoL dimensions in both professional groups. Compared with physicians, nurses reported higher mental health (62.52 vs. 58.11, *p* < 0.05), social functioning (67.17 vs. 58.96, *p* < 0.05), and overall job satisfaction (3.23 vs. 2.98, *p* < 0.01), whereas physicians scored higher in physical functioning (83.47, *p* < 0.05) and bodily pain (72.87, *p* < 0.05). **Conclusions:** Several organizational characteristics were associated with wellbeing among nurses and physicians. Protocol-based work showed consistent associations with better HRQoL and job satisfaction across multiple outcomes. However, longitudinal studies are needed to determine whether these associations reflect causal relationships.

## 1. Introduction

Nurses and physicians’ health-related quality of life (HRQoL) and job satisfaction are increasingly recognized as important indicators of workforce wellbeing, healthcare system sustainability, and quality of care [1]. Poor HRQoL and low job satisfaction have been associated with burnout, absenteeism, turnover intentions, reduced work engagement, and adverse organizational outcomes [2,3]. Identifying workplace factors associated with workforce resilience has therefore become an important priority for healthcare organizations [4,5].

The Job Demands–Resources (JD-R) model provides a comprehensive theoretical framework for understanding occupational wellbeing among nurses and physicians. According to the model, employee wellbeing is determined by the dynamic balance between job demands and job resources [6]. Job demands, including excessive workload, emotional strain, role conflict, and uncertainty, require sustained physical or psychological effort and may lead to exhaustion and impaired wellbeing [3]. Conversely, job resources facilitate goal attainment, reduce the physical and psychological costs associated with demanding work, and promote motivation, engagement, and job satisfaction [7]. Within healthcare organizations, organizational resources such as supportive leadership, effective communication, adequate staffing, structured training, and clear clinical procedures have consistently been associated with more favorable occupational outcomes [8].

Protocol-based work may be conceptualized as a potential organizational job resource within the JD-R framework. Standardized clinical protocols can provide structure for clinical decision-making, enhance role clarity, facilitate interdisciplinary communication, and promote consistency of patient care [5]. These characteristics may be relevant to perceived cognitive workload and confidence in clinical practice. Accordingly, protocol-based work may be associated with HRQoL and job satisfaction, particularly during periods characterized by heightened clinical uncertainty such as the COVID-19 pandemic. In the present study, protocol-based work refers to clinical practice primarily guided by formal clinical protocols, institutional guidelines, and standardized operating procedures implemented during the COVID-19 pandemic.

Job satisfaction reflects employees’ overall evaluation of their work experience and professional environment and has been associated with improved performance and organizational commitment [9]. Similarly, HRQoL provides a multidimensional assessment of physical, mental, and social wellbeing and is widely used to evaluate the impact of occupational conditions on healthcare professionals [1]. Understanding factors associated with both HRQoL and job satisfaction remain important for healthcare workforce sustainability [1,4].

The COVID-19 pandemic represented an unprecedented challenge for healthcare systems worldwide [10]. Nurses and physicians were exposed to increased workloads, occupational risks, rapidly changing procedures, and considerable uncertainty, resulting in substantial psychological burden and reductions in wellbeing [11,12]. Although the acute phase of the pandemic has ended, it provides a valuable context for examining factors associated with workforce wellbeing under conditions of sustained organizational pressure [6]. Furthermore, organizational support and preparedness have been associated with better wellbeing and higher job satisfaction among nurses and physicians during crisis situations [8,13]. The Greek healthcare system provides a relevant context for examining these issues. Following a prolonged period of fiscal constraints, public hospitals entered the pandemic with structural limitations that further shaped workforce conditions during the crisis [14,15]. Understanding the organizational factors associated with nurses and physicians’ wellbeing in this setting may inform workforce-support strategies applicable both during future public health emergencies and routine healthcare practice.

Although a substantial body of literature has examined burnout, psychological distress, resilience, organizational support, and job satisfaction among nurses and physicians during the COVID-19 pandemic [16], considerably less attention has been paid to protocol-based work as a distinct organizational characteristic. Previous studies have primarily investigated workload, staffing adequacy, leadership, personal protective equipment, and organizational support [17], whereas evidence regarding the association between standardized protocol-based work and healthcare professionals’ HRQoL and job satisfaction remains limited.

The present study addresses this gap by examining protocol-based work within the JD-R framework and by considering standardized clinical procedures as a potentially relevant organizational characteristic in a high-demand healthcare context. The theoretical contribution lies in extending the application of the JD-R framework to an organizational characteristic that has received comparatively limited attention in relation to healthcare professionals’ HRQoL and job satisfaction. By examining both outcomes simultaneously and comparing nurses and physicians working within the same hospital context, the study also assesses whether the observed associations are consistent across professional groups. Importantly, the study does not assume that protocol-based work constitutes an established job resource; rather, it examines whether its reported presence is associated with workforce wellbeing, thereby providing empirical evidence for further examination of this organizational characteristic within the JD-R framework.

Therefore, the present study aimed to compare HRQoL and job satisfaction between nurses and physicians working in Greek public hospitals and to examine whether protocol-based work and other workplace characteristics were independently associated with these outcomes. Based on the JD-R framework, we hypothesized that protocol-based work, conceptualized as a potential organizational job resource, would be positively associated with HRQoL and job satisfaction.

## 2. Materials and Methods

### 2.1. Study Design and Setting

A multicenter observational study with a cross-sectional design was conducted in 11 public hospitals affiliated with the 6th Regional Health Authority of Greece between September 2022 and March 2023. Although the acute phase of the COVID-19 pandemic had subsided, participating hospitals continued to operate dedicated COVID-19 units and infection-control procedures. The study was designed to examine associations between workplace characteristics, health-related quality of life (HRQoL), and job satisfaction among nurses and physicians. The manuscript was prepared according to the Strengthening the Reporting of Observational Studies in Epidemiology (STROBE) guidelines.

### 2.2. Participants and Sampling

A non-probability convenience sampling strategy was employed because recruitment depended on administrative approval from participating hospitals and staff availability during the pandemic recovery period. Consequently, probability of sampling was not feasible. Eligible participants were registered nurses and physicians employed in the participating hospitals who were actively working during the study period and provided written informed consent. Nurses and physicians who were absent because of leave or other reasons were excluded.

Eleven of the 29 eligible hospitals within the 6th Regional Health Authority agreed to participate. These hospitals represented both secondary and tertiary public healthcare facilities serving urban and semi-urban populations. Recruitment was conducted across several clinical departments, including COVID-19 units and non-COVID inpatient wards, to maximize the diversity of workplace environments represented in the study.

Because hospital participation depended on administrative approval rather than random selection, the sample should not be considered nationally representative of Greek nurses and physicians. Therefore, the findings should be interpreted as reflecting associations within the participating hospitals rather than estimates of national prevalence.

### 2.3. Power Analysis

A priori statistical power analysis was performed using G*Power V 3.1.9.7 before participant recruitment. The calculation was informed by a pilot study conducted in the same setting (92 nurses and 78 physicians). One of the primary analytical considerations was the Pearson correlation coefficient. With a Type I error rate (α) of 0.05 and statistical power of at least 80%, a minimum of 190 participants per professional group was required to detect a correlation of r = 0.25. For larger effects (r ≥ 0.30), a smaller sample size would have been sufficient; therefore, the more conservative target of 190 participants per professional group was adopted. For the planned multivariable regression analyses, an additional power calculation was performed assuming a medium effect size (f^2^ = 0.15), α = 0.05, and 80% power. Based on a model including 13 predictors, the minimum required sample size was 139 participants. Recruitment continued until the prespecified target of approximately 190 participants per professional group was reached. The final analytical sample consisted of 405 participants, including 214 nurses and 191 physicians.

### 2.4. Data Collection and Measures

Data was collected using anonymous, self-administered questionnaires. Questionnaires included: (i) sociodemographic and occupational characteristics and (ii) validated measures of health-related quality of life (HRQoL) and job satisfaction.

#### 2.4.1. Sociodemographic and Occupational Characteristics

Demographic variables included age, sex, marital status, educational level, place of residence, and household income. Occupational characteristics included years of professional experience, employment status, weekly working hours, and workplace assignment. Variables related to pandemic working conditions were also collected, including employment in a COVID-19 unit, night-shift frequency (nurses), on-call duties (physicians), and protocol-based work.

Protocol-based work was assessed using a single self-reported dichotomous item asking participants whether their routine clinical practice during the COVID-19 pandemic was primarily guided by established clinical protocols, institutional guidelines, or standardized operating procedures (Yes/No). The purpose of this variable was to capture participants’ overall perception of the extent to which standardized clinical protocols guided their daily practice. Although this approach facilitated questionnaire completion during a demanding clinical period, a single dichotomous self-reported item cannot capture the multidimensional nature of protocol implementation, including the availability, comprehensiveness, adherence to, and perceived usefulness of clinical protocols. Therefore, protocol-based work should be interpreted as a broad indicator of participants perceived exposure to standardized clinical procedures rather than as a comprehensive measure of protocol implementation or adherence.

#### 2.4.2. Health−Related Quality of Life

HRQoL was assessed using the 36-Item Short Form Health Survey (SF-36). The instrument comprises eight domains: physical functioning, role limitations due to physical health, role limitations due to emotional problems, vitality, mental health, social functioning, bodily pain, and general health. The Greek version of the SF-36 has been validated for use in the Greek population. Domain scores were calculated according to RAND scoring guidelines and transformed to a 0–100 scale, with higher scores indicating better perceived health status.

#### 2.4.3. Job Satisfaction

Job satisfaction was assessed using the Minnesota Satisfaction Questionnaire–Short Form (MSQ-Short Form). The validated Greek version (Gr-MSQ-Short) consists of 20 items rated on a five-point Likert scale ranging from 1 (“very dissatisfied”) to 5 (“very satisfied”). Intrinsic satisfaction was calculated as the mean score of 12 items, extrinsic satisfaction as the mean score of six items, and overall satisfaction as the mean score of all 20 items. Higher scores indicate greater job satisfaction.

### 2.5. Potential Confounding

Because the study focused primarily on workplace characteristics, several potentially important individual-level variables—including burnout, perceived stress, previous mental health conditions, personality characteristics, and family-related factors—were not measured. Similarly, potentially relevant organizational-level variables, including supervisory or line-manager support, workload, leadership style, staffing adequacy, organizational climate, safety climate, and organizational culture, were not assessed. These unmeasured individual- and hospital-level characteristics may have influenced both exposure to protocol-based work and the outcomes examined; therefore, residual confounding cannot be excluded.

### 2.6. Statistical Analysis

Data was analyzed using IBM SPSS Statistics version 28. Descriptive statistics were used to summarize participant characteristics and study variables. Continuous variables are presented as means and standard deviations, whereas categorical variables are presented as frequencies and percentages. Internal consistency of the study instruments was assessed using Cronbach’s alpha coefficients. Group differences were examined using independent-samples *t*-tests and one-way analysis of variance (ANOVA) with Bonferroni post hoc comparisons where appropriate. Pearson correlation coefficients were calculated to assess associations between continuous variables. Multivariable linear regression analyses were performed separately for nurses and physicians to identify factors independently associated with job satisfaction and HRQoL outcomes. Variables demonstrating statistical significance in univariable analyses were entered into the multivariable models. Model fit was evaluated using R^2^ and adjusted R^2^ values. Statistical significance was set at *p* < 0.05.

The dataset contained no missing values for any of the variables included in the analyses; therefore, all reported categories sum to the full sample size within each group. Multiple statistical comparisons were performed across several HRQoL domains and job satisfaction outcomes. No formal adjustment for multiplicity was applied; consequently, the possibility of inflated Type I error cannot be excluded. All analyses were therefore considered exploratory and hypothesis-generating rather than confirmatory. Statistically significant findings were interpreted in conjunction with effect sizes, consistency across models, and clinical relevance rather than statistical significance alone, and should not be regarded as definitive.

Participants were nested within 11 hospitals, and hospital-level clustering was not explicitly modelled. Consequently, the independence of observations cannot be fully assumed, and standard errors may have been underestimated. Differences between hospitals in staffing, resources, leadership, organizational climate, management practices, and implementation of clinical protocols may also have contributed to the observed associations. Because the number of participating hospitals was limited, a multilevel analysis was not performed. Future studies including a larger number of hospitals, should use multilevel models with hospital-level random effects to account for clustering and to examine the contribution of hospital-specific characteristics.

### 2.7. Ethical Procedures

The study was conducted in accordance with the Declaration of Helsinki and applicable data protection regulations (General Data Protection Regulation, GDPR). Ethical approval was obtained from the Research Ethics and Deontology Committee of the University of Patras (Protocol No. 12677/25-01-2022) and the 6th Regional Health Authority of Greece (Protocol No. 39834/13-09-2022). All participants provided written informed consent prior to participation. Participation was voluntary, questionnaires were completed anonymously, and access to study data was restricted to the research team.

## 3. Results

### 3.1. Participant Characteristics (n = 405)

A total of 405 healthcare professionals participated in the study, including 214 nurses (52.8%) and 191 physicians (47.2%). Participant characteristics are summarized in Table 1. Nurses were slightly older than physicians and had more professional experience. Protocol-based work during the pandemic was reported by 66.8% of nurses and 59.7% of physicians. Nearly one-half of physicians and one-third of nurses worked in dedicated COVID-19 units. As expected, nurses more frequently reported night-shift work, whereas physicians reported frequent on-call duties. Overall, the sample represented a broad range of demographic and workplace characteristics across participating hospitals.

### 3.2. Reliability of the Instruments

Table 2 presents the internal consistency of the study instruments. The SF-36 total-scale Cronbach’s α was 0.94 among nurses and 0.92 among physicians, while the corresponding values for the MSQ-Short Form were 0.90 and 0.89, respectively. At the subscale level, Cronbach’s α values for the eight SF-36 domains ranged from 0.73 to 0.89 among nurses and from 0.71 to 0.89 among physicians. For the two MSQ-Short Form dimensions, Cronbach’s α ranged from 0.77 to 0.87 among nurses and from 0.73 to 0.83 among physicians. Overall, the subscale-level coefficients indicated acceptable to good internal consistency across the study measures in both professional groups.

### 3.3. Health-Related Quality of Life and Job Satisfaction Among Nurses and Physicians

HRQoL and job satisfaction scores are presented in Table 3. Overall, both nurses and physicians reported higher physical functioning scores than general health and vitality scores. Nurses generally reported higher job satisfaction than physicians, particularly for extrinsic satisfaction (3.00 ± 0.69 vs. 2.58 ± 0.67), whereas physicians reported higher physical functioning (83.47 ± 19.08 vs. 77.44 ± 22.28). Differences in the remaining HRQoL domains were modest.

### 3.4. Comparison of HRQoL and Job Satisfaction Between Nurses and Physicians

Comparisons between nurses and physicians are presented in Table 4. Although statistically significant differences were observed for several outcomes, most effect sizes were small. The largest difference was found for extrinsic job satisfaction (Cohen’s d = −0.61, *p* < 0.001), with nurses reporting higher scores than physicians. Total job satisfaction was also higher among nurses than physicians (t(403) = −4.64, *p* < 0.001, Cohen’s d = −0.46). Nurses also demonstrated better mental health and social functioning, whereas physicians reported better physical functioning and lower bodily pain.

### 3.5. Work and Crisis−Related Differences in Nurses and Physicians

Among nurses, protocol-based work emerged as the workplace characteristic most consistently associated with better HRQoL and job satisfaction (Table 5). Nurses reporting protocol-based work had significantly higher vitality, mental health, general health, and intrinsic and extrinsic job satisfaction scores (all *p* < 0.05), with small-to-moderate effect sizes (Cohen’s d = 0.31–0.69).

Overall, protocol-based work showed more consistent associations with nurses’ wellbeing than workload-related characteristics.

### 3.6. Physicians

Among physicians, protocol-based work again emerged as the workplace characteristic most consistently associated with better HRQoL and job satisfaction (Table 6). Physicians reporting protocol-based work had significantly higher scores across several HRQoL domains and both job satisfaction dimensions (all *p* ≤ 0.007), with the largest effect sizes observed for vitality (Cohen’s d = 0.74) and job satisfaction (Cohen’s d = 0.55–0.60).

Overall, these findings indicate that organizational characteristics showed more consistent associations with physicians’ wellbeing than workload-related factors.

### 3.7. Multivariable Regression Analyses

Multivariable regression analyses (Table 7) identified protocol-based work as the organizational variable showing the most consistent associations with favorable outcomes. It remained independently associated with six of the nine outcomes examined among physicians and four of the nine outcomes examined among nurses. Among physicians, protocol-based work was independently associated with higher total job satisfaction, fewer role limitations due to physical health and emotional problems, and higher vitality, mental health, and social functioning scores (standardized β ranging from −0.19 to −0.35). Conversely, assignment to COVID-19 units was associated with poorer psychological well-being. Among nurses, protocol-based work remained significantly associated with total job satisfaction and with role limitations due to physical health, social functioning, and general health (standardized β ranging from −0.16 to −0.28), whereas older age, rural residence, and more frequent night shifts were associated with poorer general health. Because the predictors were measured on different scales, comparisons of effect magnitude were based on the standardized coefficients reported in Table 7.

## 4. Discussion

This study examined the associations between protocol-based work, workplace characteristics, HRQoL, and job satisfaction among nurses and physicians employed in Greek public hospitals. Protocol-based work was associated with several HRQoL and job satisfaction outcomes in both professional groups, whereas working in COVID-19 units, longer working hours, and night-shift work were associated with less favorable outcomes in selected analyses. These findings suggest that workplace characteristics may be relevant to workforce wellbeing during periods of healthcare crisis.

The lower vitality and general health scores observed in both nurses and physicians are consistent with previous evidence documenting substantial psychological and occupational burden among healthcare professionals during the COVID-19 pandemic, including anxiety, emotional distress, and burnout [11,12,18]. Differences between nurses and physicians were also observed across several HRQoL and job satisfaction domains. Nurses reported higher mental health, social functioning, and intrinsic and extrinsic job satisfaction, whereas physicians reported better physical functioning and lower bodily pain. These differences may reflect variations in professional roles and working conditions during the pandemic, although workload, exposure to COVID-19 units, and other unmeasured organizational factors may also have contributed to the observed patterns [19,20]. However, these potential explanations were not directly measured or tested in the present study and should therefore be interpreted cautiously.

A particularly important finding was the consistent association between protocol-based work and both HRQoL and job satisfaction across nurses and physicians. Protocol-based work was associated with multiple HRQoL domains as well as intrinsic and extrinsic job satisfaction, distinguishing it from several demographic and occupational characteristics examined in the study. From the perspective of the Job Demands–Resources (JD-R) model, protocol-based work may represent an organizational job resource by providing greater clarity and structure in complex clinical environments, particularly during periods of clinical uncertainty such as the COVID-19 pandemic. However, these findings should be interpreted as associations rather than causal effects. Hospitals with stronger organizational cultures, better leadership, staffing adequacy, or institutional support may have been more likely both to implement standardized protocols and to report higher staff wellbeing [21]. Because these factors were not directly measured, residual confounding cannot be excluded. Nevertheless, the consistency of the associations across professional groups supports further investigation of protocol-based work as a potentially relevant organizational characteristic.

Workload-related factors were also associated with poorer outcomes. Among nurses, more frequent night shifts were associated with poorer general health and lower intrinsic job satisfaction, consistent with evidence linking night work and sleep disruption to less favorable physical and psychological wellbeing [22]. Among physicians, longer weekly working hours and more frequent on-call duties were associated with lower extrinsic job satisfaction, consistent with evidence that intensive work schedules may be associated with less favorable perceptions of organizational sustainability and fairness [23,24].

Physicians working in COVID-19 internal medicine units also reported poorer HRQoL across several domains. These units represented high-intensity clinical environments characterized by sustained exposure risk, emotional burden, and continuous clinical pressure. Similar findings have been reported in Greek healthcare settings, where burnout among COVID-19 frontline staff has been associated with demanding workloads, shift patterns, and job dissatisfaction [25]. However, differences in leadership, staffing adequacy, safety climate, and hospital-level management practices may also have contributed to these differences. Thus, the findings should be interpreted as differences in wellbeing associated with particular work conditions rather than as evidence of causal effects.

### 4.1. Organizational and Crisis Management Implications

The findings suggest that organizational processes, including protocol implementation, staffing, leadership, workload, and supervisory support, may be relevant to workforce wellbeing during health crises. However, the observed associations should not be interpreted as evidence of causal effects. Future longitudinal and intervention studies using multidimensional measures of protocol implementation are needed to determine whether protocol-based work contributes to workforce wellbeing and under what organizational conditions.

### 4.2. Strengths, Limitations, and Future Research

This study has several strengths. It employed parallel measurement across nurses and physicians within the same hospital environment, enabling direct comparison of HRQoL and job satisfaction between professional groups during the pandemic. The use of validated instruments enhances comparability with previous studies, and internal consistency in the present sample was acceptable to good across all subscales (Table 2). To our knowledge, this is among the first studies to evaluate protocol-based work simultaneously in nurses and physicians using validated measures of both HRQoL and job satisfaction.

Several limitations should be acknowledged. The cross-sectional design precludes causal inference, and associations between protocol-based work and outcomes may reflect broader organizational characteristics, such as leadership quality or safety culture. Convenience sampling and self-reported measures introduce potential selection and reporting bias. Data collection occurred during a later phase of the pandemic response, when organizational practices may have partially stabilized. In addition, recruitment from hospitals within a single Regional Health Authority limits generalizability. Participants were nested within 11 hospitals that may have differed in staffing, resources, and organizational culture. Hospital-level clustering was not modelled; therefore, standard errors may have been underestimated, and residual organizational confounding cannot be excluded.

Furthermore, protocol-based work was assessed using a single self-reported dichotomous item. Although this approach provided a feasible measure during a demanding clinical period, it does not capture the multidimensional nature of protocol implementation, including protocol availability, comprehensiveness, frequency of use, adherence, and perceived usefulness. This limitation may have reduced the construct validity and precision of the exposure measure. Future studies should use validated multidimensional instruments or multiple indicators to assess protocol-based work more comprehensively.

The study did not measure burnout, perceived stress, baseline mental health, leadership quality, organizational climate, or safety culture, all of which may influence HRQoL and job satisfaction. Finally, because multiple statistical tests were performed without formal adjustment for multiplicity, some statistically significant findings may represent Type I error and should therefore be interpreted cautiously.

Future research should use longitudinal and multilevel designs to examine the temporal and organizational determinants of workforce wellbeing and to account for clustering within hospitals. Multidimensional measures of protocol implementation, together with organizational variables such as leadership, staffing, safety culture, and workload, would help clarify the mechanisms underlying the observed associations. Qualitative and cross-country comparative studies could further explore how organizational and health-system contexts shape workforce wellbeing during health crises.

## 5. Conclusions

Both nurses and physicians working in Greek public hospitals during the COVID-19 pandemic reported less favorable wellbeing in selected HRQoL domains, particularly vitality and general health. Several workplace characteristics were associated with HRQoL and job satisfaction, with protocol-based work showing relatively consistent associations across multiple outcomes in both professional groups. These findings should be interpreted as associative rather than causal, given the cross-sectional design, the single-item assessment of protocol-based work, and the presence of unmeasured individual- and hospital-level factors. Protocol-based work may represent a potentially relevant organizational characteristic, but the observed associations require further verification using validated multidimensional measures, longitudinal designs, and analyses that account for hospital-level clustering. Such studies are needed to clarify whether, and under what organizational conditions, protocol-based work is associated with workforce wellbeing.

## Figures and Tables

**Table 1:** Participant characteristics.

Characteristic	Nurses (n = 214)	Physicians (n = 191)
Age, mean (SD), years	43.31 (9.14)	41.59 (9.45)
Years of experience in hospital, mean (SD), years	15.32 (10.32)	11.95 (8.63)
Years of experience in current unit, mean (SD), years	6.96 (7.18)	6.13 (6.25)
Female, n (%)	160 (74.8%)	117 (61.3%)
Male, n (%)	54 (25.2%)	74 (38.7%)
Protocol-based work: Yes	143 (66.8%)	114 (59.7%)
Weekly working hours < 40, n (%)	36 (16.8%)	66 (34.6%)
Weekly working hours 40–59, n (%)	161 (75.2%)	77 (40.3%)
Weekly working hours ≥ 60, n (%)	17 (7.9%)	48 (25.1%)
Night shifts/month 0, n (%)	38 (17.8%)	—
Night shifts/month 1–5, n (%)	87 (40.7%)	—
Night shifts/month 6–15, n (%)	89 (41.6%)	—
On-duty calls/month 0, n (%)	—	36 (18.8%)
On-duty calls/month 1–5, n (%)	—	46 (24.1%)
On-duty calls/month 6–15, n (%)	—	109 (57.1%)
Work in COVID-19 unit: Yes, n (%)	67 (31.3%)	95 (49.7%)
Work in COVID-19 unit: No, n (%)	147 (68.7%)	96 (50.3%)

Notes: n, number of participants; %, percentage; SD, Standard Deviation; no data was missing for any variable shown.

**Table 2:** Internal consistency of study instruments.

Instrument	Group	Dimension/Subscale	Items (n)	Cronbach’s α
SF-36	Nurses	Total scale	36	0.94
SF-36	Nurses	Physical functioning	10	0.89
SF-36	Nurses	Role limitations—physical health	4	0.85
SF-36	Nurses	Role limitations—emotional problems	3	0.78
SF-36	Nurses	Vitality (energy/fatigue)	4	0.76
SF-36	Nurses	Mental health	5	0.74
SF-36	Nurses	Social functioning	2	0.73
SF-36	Nurses	Bodily pain	2	0.87
SF-36	Nurses	General health	5	0.78
MSQ−short	Nurses	Total scale	20	0.90
MSQ−short	Nurses	Intrinsic satisfaction	12	0.87
MSQ−short	Nurses	Extrinsic satisfaction	6	0.77
SF-36	Physicians	Total scale	36	0.92
SF-36	Physicians	Physical functioning	10	0.89
SF-36	Physicians	Role limitations—physical health	4	0.86
SF-36	Physicians	Role limitations—emotional problems	3	0.71
SF-36	Physicians	Vitality (energy/fatigue)	4	0.75
SF-36	Physicians	Mental health	5	0.77
SF-36	Physicians	Social functioning	2	0.72
SF-36	Physicians	Bodily pain	2	0.84
SF-36	Physicians	General health	5	0.71
MSQ−short	Physicians	Total scale	20	0.89
MSQ−short	Physicians	Intrinsic satisfaction	12	0.83
MSQ−short	Physicians	Extrinsic satisfaction	6	0.73

Notes: SF-36, Short Form-36; MSQ-short, Minnesota Satisfaction Questionnaire–Short.

**Table 3:** SF-36 (range 0–100) and MSQ-Short (range 1–5) scores by professional group (mean ± SD).

Measure	Nurses (n = 214)	Physicians (n = 191)
SF-36 Physical Functioning	77.44 ± 22.28	83.47 ± 19.08
SF-36 Role limitations—physical	67.05 ± 38.54	59.55 ± 41.08
SF-36 Role limitations—emotional	69.47 ± 38.25	64.04 ± 37.60
SF-36 Vitality (energy/fatigue)	57.24 ± 17.61	53.92 ± 16.93
SF-36 Mental health	62.52 ± 17.05	58.11 ± 17.32
SF-36 Social functioning	67.17 ± 22.53	58.96 ± 21.43
SF-36 Bodily pain	66.17 ± 25.50	72.87 ± 22.37
SF-36 General health	56.58 ± 19.81	53.42 ± 16.32
MSQ-Short Intrinsic satisfaction (1–5)	3.39 ± 0.58	3.27 ± 0.50
MSQ-Short Extrinsic satisfaction (1–5)	3.00 ± 0.69	2.58 ± 0.67
MSQ-Short Total satisfaction (1–5)	3.23 ± 0.56	2.98 ± 0.52

**Table 4:** Significant differences in HRQoL and job satisfaction between physicians and nurses.

Outcome	Physicians Mean (SD)	Nurses Mean (SD)	*t*-Test (df)	*p*-Value	Cohen’s d
Physical Functioning	83.47 (19.08)	77.44 (22.28)	t(403) = 2.91	0.004	0.29
Mental Health	58.11 (17.32)	62.52 (17.05)	t(403) = −2.58	0.010	−0.26
Social Functioning	58.96 (21.43)	67.17 (22.53)	t(403) = −3.74	<0.001	−0.37
Bodily Pain	72.87 (22.37)	66.17 (25.50)	t(403) = 2.79	0.006	0.28
Intrinsic Satisfaction (1–5)	3.27 (0.50)	3.39 (0.58)	t(403) = −2.04	0.042	−0.20
Extrinsic Satisfaction (1–5)	2.58 (0.67)	3.00 (0.69)	t(403) = −6.10	<0.001	−0.61
Total Satisfaction (1–5)	2.98 (0.52)	3.23 (0.56)	t(403) = −4.64	<0.001	−0.46

Note. SD = standard deviation. Positive Cohen’s d (physicians–nurses) favors physicians.

**Table 5:** Factors associated with HRQoL and job satisfaction among nurses.

Outcome	Comparison Groups	Group Means	Test (df)	*p*-Value	Cohen’s d
Vitality	Protocol-based work: Yes vs. No	59.06 vs. 53.59	t(212) = 2.15	0.033	0.31
Mental health	Protocol-based work: Yes vs. No	64.28 vs. 58.98	t(212) = 2.16	0.032	0.31
General health	Protocol-based work: Yes vs. No	59.06 vs. 51.62	t(212) = 2.62	0.009	0.38
Intrinsic satisfaction	Protocol-based work: Yes vs. No	3.51 vs. 3.13	t(212) = 4.75	<0.001	0.69
Extrinsic satisfaction	Protocol-based work: Yes vs. No	3.09 vs. 2.82	t(212) = 2.69	0.008	0.39
Role limitations—physical	Contract: Perm (n = 163) vs. Temp (n = 51)	64.26 vs. 75.98	t(212) = −1.91	0.057	−0.31
Vitality	Contract: Perm vs. Temp	55.83 vs. 61.76	t(212) = −2.12	0.035	−0.34
Mental health	Night shifts/month: 0 (n = 38) vs. 1–5 (n = 87) vs. 6–15 (n = 89)	69.05 vs. 60.73 vs. 61.48	F(2,211) = 3.51	0.032	–
General health	Night shifts/month: 0 vs. 1–5 vs. 6–15	63.03 vs. 58.33 vs. 52.13	F(2,211) = 4.75	0.010	–
Intrinsic satisfaction	Night shifts/month: 0 vs. 1–5 vs. 6–15	3.61 vs. 3.39 vs. 3.29	F(2,211) = 3.89	0.022	–

Note: *p*−values are two−sided; Cohen’s d is signed as (first group—second group).

**Table 6:** Factors associated with HRQoL and job satisfaction among physicians.

Outcome	Comparison Groups	Group Means	Test (df)	*p*-Value	Cohen’s d
Role limitations—physical	Protocol-based work: Yes vs. No	67.32 vs. 48.05	t(189) = 3.26	0.001	0.48
Role limitations—emotional	Protocol-based work: Yes vs. No	70.46 vs. 54.54	t(189) = 2.92	0.004	0.43
Vitality	Protocol-based work: Yes vs. No	58.68 vs. 46.88	t(189) = 5.02	<0.001	0.74
Mental health	Protocol-based work: Yes vs. No	62.17 vs. 52.10	t(189) = 4.10	<0.001	0.60
Social functioning	Protocol-based work: Yes vs. No	62.39 vs. 53.89	t(189) = 2.73	0.007	0.40
Intrinsic satisfaction	Protocol-based work: Yes vs. No	3.38 vs. 3.12	t(189) = 3.72	<0.001	0.55
Extrinsic satisfaction	Protocol-based work: Yes vs. No	2.74 vs. 2.35	t(189) = 4.09	<0.001	0.60
Vitality	COVID-19 unit (n = 95) vs. Other units (n = 96)	49.53 vs. 58.28	t(189) = −3.69	<0.001	−0.53
Mental health	COVID-19 unit vs. Other units	53.22 vs. 62.96	t(189) = −4.04	<0.001	−0.58
Social functioning	COVID-19 unit vs. Other units	53.81 vs. 64.06	t(189) = −3.39	<0.001	−0.49
Intrinsic satisfaction	COVID-19 unit vs. Other units	3.15 vs. 3.41	t(189) = −3.79	<0.001	−0.55
Extrinsic satisfaction	Weekly hours: 20–39 vs. 40–59 vs. ≥60	2.69 vs. 2.71 vs. 2.25	F(2,188) = 8.48	<0.001	–
Extrinsic satisfaction	On-duty calls: 0 vs. 1–5 vs. 6–15	2.69 vs. 2.86 vs. 2.44	F(2,188) = 7.33	<0.001	–

Note: *p*−values are two−sided; Cohen’s d is signed as (first group—second group).

**Table 7:** Independent predictors of HRQoL and job satisfaction among physicians and nurses.

Professional Group	Outcome	Significant Predictors	B (β)	SE	*p*-Value	(Adj R^2^)
Physicians	Total Job Satisfaction	Education level	0.259 (0.365)	0.047	<0.001	0.19
		Protocol-based work	−0.277 (−0.289)	0.070	<0.001	
		COVID-19 unit	0.159 (0.169)	0.064	0.014	
Physicians	Role Limitations (Physical)	Age	−2.250 (−0.517)	0.567	<0.001	0.21
		Protocol-based work	−24.929 (−0.298)	6.329	<0.001	
		Marital status	16.756 (0.204)	6.628	0.012	
		COVID-19 unit	14.178 (0.173)	5.835	0.016	
Physicians	Role Limitations (Emotional)	Age	−2.567 (−0.645)	0.522	<0.001	0.16
		Marital status	19.754 (0.263)	6.104	0.001	
		COVID-19 unit	15.877 (0.212)	5.374	0.004	
		Protocol-based work	−18.753 (−0.245)	5.829	0.002	
Physicians	Vitality	Protocol-based work	−11.972 (−0.348)	2.558	<0.001	0.23
		COVID-19 unit	9.008 (0.267)	2.358	<0.001	
Physicians	Mental Health	COVID-19 unit	9.624 (0.278)	2.511	<0.001	0.16
		Protocol-based work	−9.051 (−0.257)	2.723	0.001	
		Education level	4.405 (0.169)	1.855	0.019	
Physicians	Social Functioning	Marital status	9.440 (0.221)	3.553	0.009	0.09
		Education level	5.965 (0.185)	2.311	0.011	
		COVID-19 unit	9.028 (0.211)	3.128	0.004	
		Protocol-based work	−8.449 (−0.194)	3.393	0.014	
Nurses	Total Job Satisfaction	Protocol-based work	−0.302 (−0.276)	0.084	0.001	0.10
Nurses	Role Limitations (Physical)	Marital status	−13.231 (−0.155)	6.630	0.047	0.08
		Protocol-based work	−13.051 (−0.160)	6.237	0.038	
Nurses	Social Functioning	Protocol-based work	−8.556 (−0.179)	3.737	0.023	0.03
Nurses	General Health	Rural residence	−6.293 (−0.159)	2.747	0.023	0.15
		Night shifts	−5.203 (−0.193)	1.933	0.008	
		Age	−0.739 (−0.341)	0.266	0.006	
		Protocol-based work	−7.839 (−0.187)	3.099	0.012	

Note: B, unstandardized coefficient; β, standardized coefficient; SE, standard error. Protocol-based work, work in a COVID-19 unit, and previous pandemic experience were reverse-coded (Yes = 0, No = 1); therefore, negative coefficients for these variables indicate higher outcome scores among participants who answered “Yes.” The table reports the statistically significant predictors from each regression model in which protocol-based work was significantly associated with the outcome. Full models including all 13 predictors are available from the corresponding author. Adjusted R^2^ is reported for the models included in the original analysis; — indicates that the value was not reported.

## Data Availability

The data presented in this study are available on request from the corresponding author. The data is not publicly available due to ethical and privacy restrictions (human participant data and institutional approvals).
